# Non-prostatic pathology on prostate needle-biopsy – colorectal carcinoid: a case report

**DOI:** 10.1186/1757-1626-2-75

**Published:** 2009-01-21

**Authors:** Roderick CN van den Bergh, Tineke Wolters, Manon CW Spaander, Fritz H Schröder, Geert JLH van Leenders

**Affiliations:** 1 Department of Urology, Erasmus University Medical Center, PO Box 2040, 3000 CA, Rotterdam, the Netherlands; 2 Department of Pathology, Erasmus University Medical Center, PO Box 2040, 3000 CA, Rotterdam, the Netherlands; 3 Department of Gastroenterology, Erasmus University Medical Center, PO Box 2040, 3000 CA, Rotterdam, the Netherlands

## Abstract

**Introduction:**

Prostate needle-biopsies are among the most common specimens in routine histopathological practice; in 15% colorectal tissue is also present. Rectal pathology is described to be found in 17% of this coincidentally obtained material.

**Case presentation:**

We present a case in which colorectal carcinoid was found in the rectal mucosa obtained via transrectal prostate biopsies in a screening program for prostate cancer in a 71-year old Caucasian male. To the best of our knowledge, this was the first time that such a coincidental finding was discovered. Besides a colonoscopy with polypectomy, this coincidental detection remained without any further clinical consequences for this patient until today.

**Conclusion:**

As there is a considerable chance that abnormalities are found in the rectal tissue of prostate biopsies, it is advisable for all pathologists to include this tissue in the histology evaluation and look for potential irregularities in this simultaneously collected material.

## Introduction

Prostate needle-biopsies are among the most common specimens in routine histopathological practice following cutaneous and gastro-intestinal biopsies. Sporadically, non-prostatic pathology is encountered in needle-biopsies representing either generalised disease such as vasculitis or tuberculosis, or loco-regional aberrations i.e. extension of bladder or colorectal carcinoma. Here, we report on a case of incidentally finding colorectal carcinoid identified at prostate biopsies. To the best of our knowledge, this has never been described in the English literature before.

## Case presentation

The 71-year-old Caucasian male subject without any comorbidity participated in the Dutch section of the European Randomized Study of Screening for Prostate Cancer (ERSPC). This study has been ongoing for almost 15 years in 8 European countries and aims to explore the feasibility of population-based screening for prostate cancer [1]. Almost 270.000 men have been randomized to the screening or control group. Prostate biopsy indications have changed during the course of the study, but include abnormal digital rectal examination (DRE) and/or transrectal ultrasound (TRUS) findings, and/or elevated levels of prostate specific antigen (PSA) in the blood serum and/or PCA3 (Prostate Cancer Gene 3) in the urine [2].

In October 2007 our patient was screened using a measurement of the PSA serum level. His PSA was with 1.2 ng/ml below the biopsy-threshold of 3.0 ng/ml. However, as this man had also consented in participating in the PCA3 side study, his urinary sample (collected after DRE) was subjected to PCA3 analysis, which resulted in a score of 13. Since a threshold of PCA3 > 10 was used as a biopsy indication, this prompted DRE, TRUS, and lateralised sextant TRUS-guided prostate biopsies [3].

**Figure 1:**
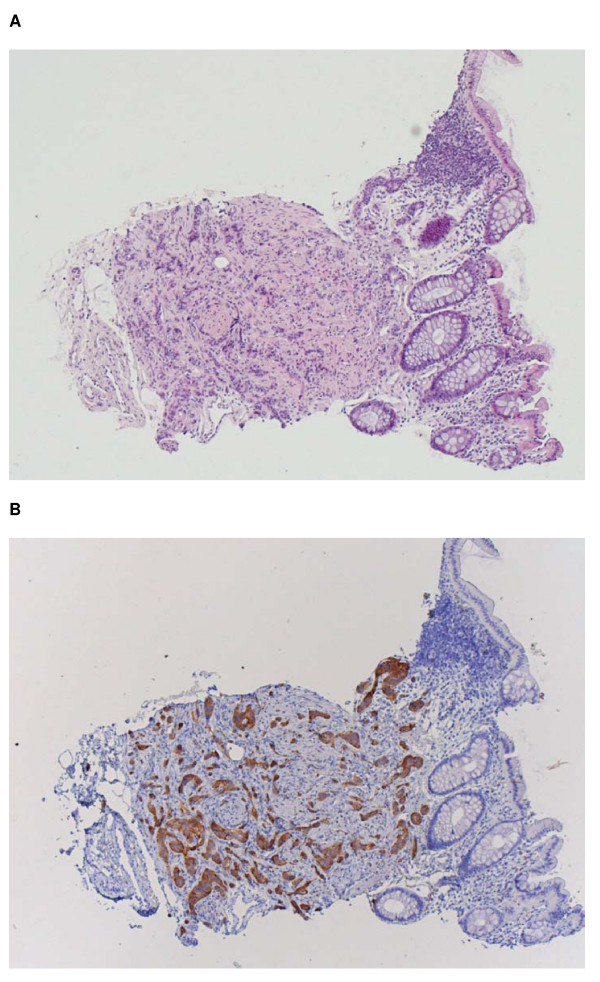
**Rectal mucosa (haematoxylin-eosin, ×40) with a carcinoid within the muscularis mucosae, characterised by a proliferation of nests, glands and strands of non-atypical epithelial cells (A), which demonstrate expression of synaptophysin (B)**.

No abnormalities were found during DRE and TRUS. Prostate volume measured by planimetric calculation was 30.9 cc. Histopathologic evaluation revealed no prostate cancer in the 6 biopsy-cores. However, in 2 of the 3 needle-biopsies that also contained rectal mucosa (number IV and V; taken from the left lateral side of the basis and middle of the prostate), a carcinoid was coincidentally found in the rectal mucosa. Small nests, glands and strands consisting of epithelial cells were present within the rectal muscularis mucosae (Figure 1). These epithelial cells were characterised by an eosinophilic, finely granular cytoplasm, a round nucleus and inconspicuous nucleolus. No significant atypia, mitotic activity or necrosis was encountered. Additional immunohistochemical stainings showed strong expression of chromogranin and synaptophysin compatible with a carcinoid/low-grade neuroendocrine tumour. Molecular investigation eliminated the option of mixing of specimens.

Our patient was informed about the benign outcome of his prostate biopsies and the remarkable finding in the rectal mucosa by the principal investigator of the ERSPC and he was advised to make an appointment with the gastroenterologist. Besides constipation, no complaints of the gastroenterologic tract or lab changes were found. A colonoscopy was performed, during which two polyps were seen, the first at 2 cm from the anus and the second at 90 cm. The carcinoid was not detected during colonoscopy, probably because it was located in the submucosa. Both polyps were removed endoscopically and sent in for pathologic examination. The lesion in the rectum was a tubulovillous adenoma with low-grade dysplasia. The second was a hyperplastic polyp without any adenomatous changes. Because of the first finding, the patient was advised to undergo a control colonoscopy after 6 years. Until today the incidental finding of carcinoid did not have any clinical consequences for this patient.

## Discussion

Carcinoid is a slow-growing but often malignant type of neuroendocrine tumour, which can arise from the enterochromaffin cells throughout different organs of the digestive and respiratory tract, and has the ability to secrete different endocrine products. In 10–20% of cases there are multiple localisations. The appendix has previously been described as the most common localisation, followed by the ileum, rectum, and stomach, but recently a marked increases in gastric and rectal carcinoids and a concomitant decrease in appendiceal carcinoid incidence has been observed. Patients with metastatic carcinoid, especially those with metastases in the liver, can show signs of the carcinoid syndrome. This is due to the production of serotonin, which is released into the systemic circulation, which leads to symptoms of cutaneous flushing, diarrhoea, bronchoconstriction and right-sided cardiac valve disease. The incidence is approximately 10–20 new clinical cases per million per year. Often these tumours however remain subclinical and are found coincidentally in resection specimens or at obduction (in 1%) [4,5].

In this case-report, prostate biopsies were the basis of the surprising finding of carcinoid. As TRUS-guided prostate biopsies perforate the wall of the rectum, biopsy core specimens may also contain a small piece of colorectal tissue. This part could potentially harbour pathology or could incur artefacts that cause diagnostic difficulty. One should especially be aware that prostate adenocarcinoma, particularly of ductal-type cancer might simulate rectal adenoma [6]. A tubulo-villous growth pattern with elongated nuclei and frequent detachment of pre-existent prostate tissue should prompt close pathologic examination eventually sustained with immunohistochemistry.

A recent retrospective article found rectal mucosa in 114 out of 782 (14.6%) reviewed prostate biopsy cores. Material from as much as 19 of these 114 (16.7%) showed relevant rectal pathology, consisting of a hyperplastic polyp in 1, changes consistent with ulcerative proctitis in 8, focal active proctitis in 7, and granulomas in 3 [7]. Incidental findings due to other investigations of the prostate besides biopsies, such as the finding of rectal carcinoma during TRUS, have also been described [8].

The patient in our report did not have any complaints of the carcinoid, neither were any lab changes found. Although having had the extra burden of the diagnosis carcinoid and a colonoscopy as the result of the screening procedure for prostate cancer, no further clinical consequences were related to this rare finding.

## Conclusion

We report on a case of the finding of colorectal carcinoid in the rectal mucosa obtained via prostate biopsies in a screening program for prostate cancer. Although this specific finding is rare, concomitant colorectal pathology in coincidentally obtained tissue at prostate biopsy is not uncommon. Histology evaluation of prostatic needle-biopsies should therefore include examination of the adjacent colorectal wall.

## Competing interests

The authors declare that they have no competing interests.

## Authors' contributions

RvdB and TW were involved in collection of data and material used during preparation of the paper and revised the manuscript critically for important intellectual content. RvdB drafted an initial version of the paper. MCWS was involved in collection of data and material used during preparation of the paper and is involved in the care of the patient. FHS is the principal investigator of the European Randomized Study of Screening for Prostate Cancer and is involved in the care of the patient. GJLHvL performed the histological examination of the prostate biopsies and is the prime supervisor of the work. All authors read and approved the final manuscript.

## Consent

Written informed consent was obtained from the patient for publication of this case report and accompanying images. A copy of the written consent is available for review by the Editor-in-Chief of this journal.
